# TCP2 positively regulates *HY5/HYH* and photomorphogenesis in Arabidopsis

**DOI:** 10.1093/jxb/erv495

**Published:** 2015-11-23

**Authors:** Zhimin He, Xiaoying Zhao, Fanna Kong, Zecheng Zuo, Xuanming Liu

**Affiliations:** ^1^College of Biology, Hunan University, Changsha 410082, China; ^2^College of Marine Life Sciences, Ocean University of China, Qingdao 266003, China; ^3^College of Plant Sciences, Jilin University, Changchun 130062, Jilin, China; ^4^State Key Laboratory of Chemo/Biosensing and Chemometrics, Hunan University, Changsha 410082, China

**Keywords:** Arabidopsis, blue light signal transduction, ChIP-qPCR, *CRY1*, *HY5*, *HYH*, hypocotyls, *TCP2*.

## Abstract

Light regulates plant growth and development via multiple photoreceptors including phytochromes and cryptochromes. Although the functions of photoreceptors have been studied extensively, questions remain regarding the involvement of cryptochromes in photomorphogenesis. In this study, we identified a protein, TEOSINTE-LIKE1, CYCLOIDEA, and PROLIFERATING CELL FACTOR 2 (TCP2), which interacts with the cryptochrome 1 (CRY1) protein in yeast and plant cells via the N-terminal domains of both proteins. Transgenic plants overexpressing *TCP2* displayed a light-dependent short hypocotyl phenotype, especially in response to blue light. Moreover, light affected TCP2 expression in a wavelength-dependent manner and TCP2 positively regulates mRNA expression of *HYH* and *HY5*. These results support the hypothesis that TCP2 is a transcription activator which acts downstream of multiple photoreceptors, including CRY1.

## Highlight

A novel CRY1-interacting protein, TCP2, regulated by blue light and involved in the CRY1-mediated blue light signal pathway to regulate photomorphogenesis, is described.

## Introduction

Arabidopsis cryptochrome (CRY) proteins are blue light receptors that mediate photomorphogenesis and photoperiodic flowering. Through blue-light-specific conformational changes, they interact with their downstream partners to modulate gene expression and alter photomorphogenesis or photoperiodic flowering (Yang *et al.*, 2000; Wang *et al.*, 2001; Sang *et al.*, 2005; Liu *et al.*, 2011; Zuo *et al.*, 2011). Numerous mechanisms by which CRYs could regulate plant development have been proposed. CRYs may mediate blue light suppression of the C*ONSTITUTIVELY PHOTOMORPHOGENIC 1* (*COP1*) gene through the CRY–SPA1/COP1 pathway, thereby affecting the expression of downstream genes. In this pathway, COP1, an E3 ubiquitin ligase, acts as a negative regulator of photomorphogenesis and photoperiodic flowering (Kim *et al.*, 2002; Ma *et al.*, 2002; Yadav *et al.*, 2002; Liu *et al.*, 2008b). COP1 interacts with SUPPRESSOR OF PHYTOCHROME A1 (SPA1) protein through the ubiquitination pathway to degrade various downstream transcription factors such as HY5 (ELONGATED HYPOCOTYL 5), HYH (HY5-HOMOLOG), HFR1 (REDUCED SENSITIVITY TO FAR-RED LIGHT 1), and CO (CONSTANS), which are required for photomorphogenesis and photoperiod flowering (Holm *et al.*, 2002; Kim *et al.*, 2002; Seo *et al.*, 2003). In Arabidopsis, CRYs interact with the COP1-interacting protein SPA1 in a blue-light-dependent manner. In blue light, SPA1 acts as a downstream regulator of CRYs to mediate the blue-light-specific suppression of the degradation of *COP1*-dependent transcription factors (Liu *et al.*, 2011; Zuo *et al.*, 2011). CRYs also regulate plant development through the CRY2–CIB1 (CRYPTOCHROME-INTERACTING BASIC–HELIX–LOOP HELIX1) pathway. In addition to modulating the gene expression level of downstream genes by suppressing the COP1–SPA1 complex, CRY2 interacts directly with the CIB1 transcription factor to regulate the transcription of downstream genes. CIB1 interacts with CRY2 in a blue-light-dependent manner and binds to the E-box contained in the *FT* promoter to regulate floral initiation in a CRY2-independent manner (Liu *et al.*, 2008a). Furthermore, CRYs may regulate downstream genes by affecting the plant circadian clock (Jarillo *et al.*, 2001).

Three CRY proteins are present in Arabidopsis: CRY1, CRY2, and CRY-DASH. Arabidopsis CRY1 was the first CRY to be identified from any organism (Ahmad and Cashmore, 1993). It mediates blue-light-dependent de-etiolation responses and regulates root growth, stomatal opening, etc. (Ahmad and Cashmore, 1996; Lin *et al.*, 1996; Ahmad *et al.*, 1998a; Ahmad, 1999; Sullivan and Deng, 2003; Tsuchida-Mayama *et al.*, 2010). In addition, CRY1 may mediate a complicated signaling network to regulate plant growth and development; however, hitherto, only SPAs were found to interact with CRY1 in a blue-light-dependent manner in this network.

In this study, we used a blue-light-differentiated yeast two-hybrid assay to screen *Arabidopsis thaliana* transcription factors that specifically interact with CRY1 and isolated a novel *CRY1*-interacting transcription factor designated TEOSINTE-LIKE1, CYCLOIDEA, and PROLIFERATING CELL FACTOR 2 (TCP2). TCP2 belongs to the Class II subfamily of the TCP family which includes 24 small proteins with a characteristic plant-specific DNA-binding domain, the TCP domain (Cubas *et al.*, 1999; Martin-Trillo and Cubas, 2010). TCP2 interacts with CRY1 in the nuclei of yeast and plants, and mediates light-regulated expression of genes involved in photomorphogenesis. Our results demonstrate a novel mechanism of CRY1 photosensory signaling.

## Materials and methods

### Plant material

*cry11-304*, *tcp2* (Col accession), *tcp2tcp4* (Col accession), *tcp2tcp4tcp10* (Col accession), *salk060818C* (homozygous TCP2 T-DNA insertion mutant, Col accession), *hy5* (Ws accession), *hy5hyh* (Ws accession), *cry1cry2*, *cry2*, *phyA*, *phyB*, *phyAphyB*, *ztl3*, *ztl3lkp2*, *cop1-4*, and *cop1-6* have been previously reported (Yang *et al.*, 1995; Mockler *et al.*, 1999; Holm *et al.*, 2002; Alonso *et al.*, 2003; Palatnik *et al.*, 2003; Nakagawa and Komeda, 2004; Sung *et al.*, 2007; Takase *et al.*, 2011). Col4, Col0, and WT (*rdr6*) (Harmoko *et al.*, 2013) were used as control or backgrounds for transformation in this study. Transgenic lines expressing 35S::*Myc-TCP2*, 35S::*LUC-TCP2*, and *TCP2RNAi* were prepared by the floral dipping method in different genetic backgrounds.

### Yeast two-hybrid assay

Yeast two-hybrid assay was performed according to the manufacturer’s instructions (Clontech Yeast Handbook).

#### Screening of CRY1-interacting transcription factor protein

The coding sequence (CDS) of *CRY1* was cloned and fused in-frame with the CDS of the GAL4 DNA-binding domain (BD) in the bait vector pDEST™32. The *CRY1*-pDEST™32 construct was transformed into the yeast strain AH109. The transformants containing *CRY1*-pDEST™32 were mated with each transcription factor gene of the Y187 yeast colony library (Pruneda-Paz *et al.*, 2014), respectively. The successfully mated yeasts grew on SD/–Trp/–Leu and were used for the subsequent yeast two-hybrid interaction tests including the auxotrophic assay and liquid assay.

#### The TCP2 domains interact with CRY1

The CDS of *CRY1* was cloned and fused in-frame with the CDS of the GAL4 BD in the bait vector pBridge. The CDS of full-length *TCP2*, *TCP* (amino acids 1–174), the *TCPn* domain (amino acids 1–150), the *R&CT* domain (amino acids 151–365), and the *CT* domain (amino acids 175–365) were cloned and fused in-frame with the CDS of the GAL4 transcription-activation domain (AD) in the prey vector pGADT7. The bait and prey plasmids were co-transformed into the yeast strain AH109.

#### The CRY1 domains interact with TCP2

The CDS of full-length *CRY1*, *CRY1N* ^*505*^ (amino acids 1–505), *CRY1N* ^*515*^ (amino acids 1–515), *CRY1N* ^*493*^ (amino acids 1–493), *CRY1M* ^*251–545*^ (amino acids 251–545), and *CRY1C* ^*301*^ (amino acids 382–682) were fused in-frame with the CDS of the GAL4 BD in the bait vector pDEST™32 (Invitrogen).The CDS of *TCP2* was fused in-frame with the CDS of the GAL4 AD in the prey vector pDEST^TM^22 (Invitrogen). The bait and prey plasmids were co-transformed into the yeast strain AH109. For the auxotrophic assay, yeast colonies were plated on SD/–Trp/–Leu and SD/–Trp/–Leu/–His/+3-aminotriazole (3-AT; 10mM) plates, and grown under blue light (35 μmol m^−2^ s^−1^), red light (18 μmol m^−2^ s^−1^), far-red light (20 μmol m^−2^ s^−1^), or in darkness at 28 °C for 3 d. The β-galactosidase (β-gal) assay was performed to quantify protein–protein interactions according to the manufacturer’s instructions, using chlorophenol red β-d-galactopyranoside (CPRG) as the substrate. Light and time treatments are indicated in the figures, and Miller units were calculated according the manufacturer’s recommendations (Clontech Yeast Handbook, Protocol # PT3024-1, version # PR742227).

### Bimolecular fluorescence complementation

Bimolecular fluorescence complementation (BiFC) was performed by using *Agrobacterium tumefaciens*-mediated transient expression in *Nicotiana benthamiana*. The CDS of *CRY1*, the different domains of *CRY1* which were used in the yeast two-hybrid assay, and *TCP2* were cloned into the pcCFP-GW or pnYFP-GW vector by using the Gateway recombination system (Meng *et al.*, 2013). The plasmids were introduced into *A. tumefaciens* strain AGL0 by electroporation. For co-infiltrations, the infiltration solution (10mM MES, pH 5.7, 10mM MgCl_2_, 5mg ml^–1^ glucose, 150 μM acetosyringone) of *Agrobacterium* with plasmid was adjusted to an OD_600_ of 0.5, and the solutions were equally mixed, and incubated for 3h at room temperature (~22–25 °C). After that, the mixed solutions were infiltrated onto the *N. benthamiana* leaves. Plants were left in a dark room overnight, and then transferred to white light for 48h; the details of the 2h light treatment which was used before fluorescence microscopy assay are indicated in the figures (Yu *et al.*, 2007). Images were captured by using a Zeiss AxioImager Z1 microscope with a Hamamatsu Orca-ER camera. The percentage of cells showing BiFC fluorescence signals was determined from the number of cells showing BiFC fluorescence signals in the nuclei; the lowest cell number showing a signal for nuclei is indicated in the figure legends for individual experiments (*n*=3).

### Co-immunoprecipitation assays

Co-immunoprecipitation analysis was performed as described (H. Liu *et al.*, 2008). Three-week-old plants grown in long days (LDs; 16h/8h) were transferred to a dark room for 16–24h, and the leaves were collected and sliced into 1mm strips, treated with 50 μM MG132 for 4h to avoid protein degradation, and then transferred to blue, red, and far-red light for the indicated times. Samples were collected and ground into a fine powder in liquid nitrogen and suspended in NEB buffer [20mM HEPES, pH 7.5, 40mM KCl, 1mM EDTA, 1% Trxton X-100, 1mM phenylmethylsulphonyl fluoride (PMSF), one tablet per 25ml protease inhibitor cocktail), using 100mg of powder with 125 μl of NEB buffer; the protein extracts were then incubated at 4 °C on a shaker for 10min, centrifuged for 15min at 16 000 *g*, and the supernatant was transferred to another new tube. Approximately 20 μl of supernatant was kept as input (total protein). The supernatants were mixed at 4 °C for 90min with 15 μl of agarose beads which had been conjugated with anti-Myc antibody (Cat#A7470, Sigma). Beads were collected by spinning down at 1000rpm for 2min and washed three times with the NEB wash buffer (20mM HEPES, pH 7.5, 40mM KCl, 0.1% Triton X-100, 1mM PMSF, one tablet per 50ml protease inhibitor cocktail). The proteins were eluted by using 4× SDS–PAGE sample buffer, boiled for 10min, and the beads were spun down at 10 000rpm for 3min at room temperature. Total supernatants were fractioned by 10% SDS–PAGE, and the membranes were probed by anti-CRY1, and then stripped, and re-probed by anti-Myc antibody (Millipore; Cat #05-724).

### Protein expression level analysis

Protein expression level analysis was performed according to a previous study (Liu *et al.*, 2013). Briefly, 3-to 4-week-old plants grown in LDs (16h/8h) were transferred to a dark room for 16–24h, and pre-treated with different light red, far-red, or blue) for the indicated times. Additionally, for MG132 treatment, 50 μM MG132 or 0.1% dimethylsulphoxide (DMSO; no MG132) were applied to pre-treat the sliced 1mm plant leaf strips in blue light (35 μmol m^−2^ s^−1^) for 4h, and then transferred to darkness for the indicated time. Samples were collected and ground into powder in liquid nitrogen. Total protein was extracted using 100 µl of extraction buffer [0.1M EDTA, pH 8.0, 0.12M TRIS-HCl, pH 6.8, 4% SDS (w/v), 10% β-mercaptoethanol (v/v), 5% glycerol (v/v), 0.005% bromophenol blue (w/v)] per 100mg of powder, boiled for 10min and then centrifuged for 10min at 14 000rpm. Proteins were fractionated by 10% SDS–PAGE and transferred to a nylon membrane for immunoblotting. Blots were probed by a mouse monoclonal anti-Myc antibody to detect Myc-TCP2 fusion protein, stripped, and then re-probed by anti-HSP90 or Ponceau staining (amounts of Rubisco) for the loading control. Immunoblot signals were quantified by ImageJ (Yu *et al.*, 2007).

### Hypocotyl and cotyledon phenotype analysis

Seedlings were grown on Murashige and Skoog (MS) medium for 5 d in different light. The cotyledon and hypocotyl phenotype were photographed; the lengths of the hypocotyls of >20 seedlings were measured. The standard deviations were calculated.

### Analysis of mRNA expression

Seedlings were grown on MS medium for 10 d under white light. For light treatment, seedlings were pre-treated in an initial light and then transferred to a different light for the indicated time before samples were collected. Total RNAs were isolated by using the GeneJET RNA Purification Kit (Thermo). The cDNA was synthesized from 1 μg of total RNA by using the Superscript first-strand cDNA synthesis system (Invitrogen). Platinum^®^SYBR^®^ Green qPCR SuperMix-UDG (Invitrogen) was used for quatitative PCR (qPCR), using the MX3000 System (Stratagene). Briefly, the cDNA was diluted 50- or 100-fold, and 2 µl of diluted cDNA was used as template in a 10 µl qPCR which was pre-denatured at 95 °C for 5min, followed by a 40 cycle program (95 °C, 10s; 58 °C, 30s; 72 °C, 30s per cycle). The mRNA level of *ACTIN2* was used as the internal control. The qPCR results shown are the average (±SD) of three biological repeats. All the primers used are described in Supplementary Table S1 available at *JXB* online.

### Subcellular localization analysis

To investigate the subcellular localization of TCP2, the CDS of *TCP2* was cloned into the vector pEarleyGate103 by using gateway technology to express TCP2–green fluorescent protein (GFP) fusion protein. The plasmid was introduced into *A. tumefaciens* strain AGL0 by electroporation. *Agrobacterium* was then infiltrated onto tobacco leaves according to the method described above, for transient expression of TCP2–GFP protein. TCP2–GFP subcellular localization was observed using a fluorescence microscope (Zeiss AxioImager Z1 microscope with a Hamamatsu Orca-ER camera).

To generate *TCP2-GFP*/wild-type (WT) transgenic lines, the plasmids were transformed into plants by the floral dip method. Four-day-old transgenic seedlings were used to detect signals of GFP stimulated by blue light under the fluorescence microscope (Zeiss AxioImager Z1 microscope with a Hamamatsu Orca-ER camera). TCP2–GFP subcellular localization is easily detected in the root without interference by chlorophyll.

Nuclear localization analysis was performed as follows: in brief, 7-day-old seedlings of *TCP2-GFP*/WT transgenic and WT plants grown on MS medium under continuous white light were collected and fixed in 10ml of TRIS buffer (10mM TRIS-HCl pH 7.5, 2mM EDTA-Na, 100mM NaCl) containing 4% (w/v) formaldehyde at room temperature for 20min. Then the seedlings were washed with cold TRIS buffer twice, each time for 10min. The seedlings were dried with filter paper and then treated with 200 µl of LB01 buffer (15mM TRIS-HCl pH 7.5, 2mM EDTA-Na, 0.5mM spermine-4HCl, 80mM KCl, 20mM NaCl, 0.1% Triton X-100). Seedlings were excised with a blade. The cell lysate subsequently was filtered through a FALCON filter tube. A 3 μl aliquot of nuclear suspension was mixed with 12 µl of sorting buffer (100mM TRIS-HCl pH 7.5, 50mM KCl, 2mM MgCl_2_, 0.05% Tween-20, 5% sucrose) on glass slides and left to air-dry for 30min. Subsequently, 10 µl of 4',6-diamidino-2-phenylindole (DAPI) solution (5 µg ml^–1^) was added to cover the dried spot and then a coverslip was placed over it. Nail polish was used to seal all around the coverslip. Images were captured by using a fluorescence microscope (Zeiss AxioImager Z1 microscope with a Hamamatsu Orca-ER camera). DAPI nuclear staining (blue), the fluorescence microscopy image of TCP2–GFP (green), and the overlay of the two images are shown.

### Chromatin immunoprecipitation quantitative PCR (ChIP-qPCR) assays

The ChIP assay was performed as described with minor modifications (Meng *et al.*, 2013). Briefly, 1g samples of 8-day-old seedlings were collected and ground into fine powder using liquid nitrogen. The powder was homogenized in nuclear isolation buffer (10mM HEPES pH 7.5, 1M sucrose, 5mM KCl, 5mM MgCl_2_, 1% formaldehyde, 14mM β-mercaptoethanol, 0.6% Triton X-100, 0.4mM PMSF) at room temperature for 10min. Glycine was added to a final concentration of 0.125M to stop cross-linking at room temperature for 5min. The homogenized slurry was filtered through two layers of Miracloth into a fresh tube placed on ice prior to precipitating the nuclei by centrifuging for 20min at 3000 *g* at 4 °C. The precipitate of the isolated nuclei was washed with two different buffers: extraction buffer 2 (0.25M sucrose, 10mM TRIS-HCl, pH 8.0, 10mM MgCl_2_, 1% Triton X-100, 5mM β- mercaptoethanol, 0.1mM PMSF, one tablet per 50ml protease inhibitor cocktail) and extraction buffer 3 (1.7M sucrose, 10mM TRIS-HCl, pH 8.0, 2mM MgCl_2_, 0.15% Triton X-100, 5mM β-mercaptoethanol, 0.1mM PMSF, one tablet per 50ml protease inhibitor cocktail). Then the precipitate of the isolated nuclei was suspended in nuclear lysis buffer (0.5ml of 1M TRIS-HCl, pH 8.0, 200 µl 0.5M EDTA, 0.5ml of 20% SDS, one tablet of protease inhibitor cocktail). The chromatin DNAs were sheared into 500bp fragments by sonication. The chromatin solution was diluted 10-fold with ChIP dilution buffer (1.1ml of 20% Triton X-100, 48 µl of 0.5M EDTA, 334 µl of 1M TRIS-HCl pH 8.0, 668 µl of 5M NaCl, 200 µl of 100mM PMSF). Anti-Myc affinity gel (Cat#SAB 4700447, Sigma) or protein A/G agarose beads were mixed with the chromatin solution and incubated at 4 °C for 3h. Immunocomplexes were washed three times with ChIP dilution buffer, and the bound chromatin fragments were eluted from beads with 500 µl of elution buffer (1% SDS, 0.1688g of NaHCO_3_ in 20ml) at 65 °C, and the cross-linking was reversed by incubating at 65 °C overnight. The mixture was treated with proteinase K for 1h at 45 °C to remove proteins. The genomic DNA was purified from the mixture using chloroform:isoamyl alcohol (24:1), and precipitated by adding 1/10vol. of 0.3M NaOAC, 2 µl of glycogen carrier (10mg ml^–1^), and 1vol. of ethanol to the supernatant. The pellet was washed with 70% ethanol and suspended in 50 µl of H_2_O. For qPCR, the DNA is further purified by using a DNA purification kit (Qiagen), diluting the DNA solution 50-fold, and then subjecting it to qPCR. The primers used are listed in Supplementary Table S1 at *JXB* online.

## Results

### TCP2 physically interacts with CRY1 in yeast and plant cells

**Fig. 1.**
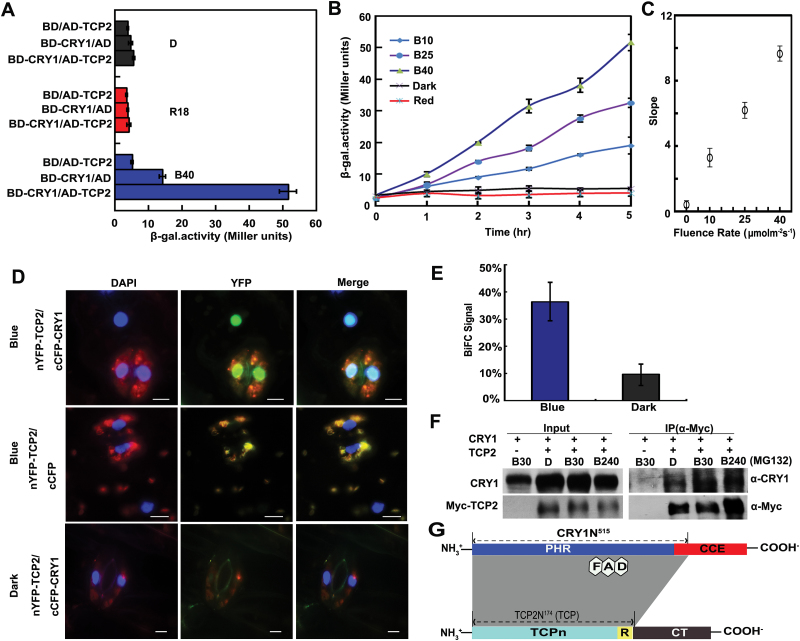
Analysis of the blue light response of the interaction between CRY1 and TCP2 in yeast cells and plant cells. (A) β-Galactosidase (β-gal) assay showing the interaction of CRY1 with TCP2 in the yeast cells treated with blue light (B 40, 40 μmol m^−2^ s^−1^), red light (R 18, 18 μmol m^−2^ s^−1^), or darkness (D) for 4h. (B) β-Gal assay showing the interaction of CRY1 with TCP2 in the yeast cells in response to blue light [10 μmol m^−2^ s^−1^ (B 10), 25 μmol m^−2^ s^−1^ (B 25), and 40 μmol m^−2^ s^−1^ (B 40)], red light (R 18, 18 μmol m^−2^ s^−1^), or darkness (D) for the indicated times. (C) Slopes of linear regression curves of different light fluence rates as shown in (B). The error bar shows the SD for triplicae samples at the same fluence rate. (D) BiFC assay showing the CRY1–TCP2 interaction in *Nicotiana benthamiana*. Plants were grown in long days (LDs; 16h/8h) for 4 weeks and the young leaves were infiltrated with an *Agrobacterium* mix carrying the plasmids nYFP-*TCP2* and cCFP-*CRY1* independently, incubated in the dark for 12h, and then transferred to white light for 48h, before fluorescence microscope assay, transferred to blue light (50 μmol m^−2^ s^−1^) and to darkness for 4h. DAPI, nuclear fluorescence; YFP, yellow fluorescent protein fluorescence; Merge, merge of DAPI and YFP; scale bar=2 μm. (E) The percentage of *N. benthamiana* leaf cells that showed the BiFC fluorescence signals in (D) were counted. For each sample at least 200 cells were counted; *P*=0.003. (F) A co-immunoprecipitation assay showing the CRY1–TCP2 interaction in blue light in Arabidopsis with the indicated treatment times. The transgenic plants expressing 35S*::Myc-TCP2*/WT were grown in LDs (16h/ 8h) for 3 weeks. Plants were transferred to darkness for 16h, and the leaves were excised, incubated in MG132 (50 μmol l^–1^) under darkness for 4h, and then exposed to blue light (B, 35 μmol m^−2^ s^−1^) for the time indicated (B 0, 0min; B 30, 30min; B 240, 240min). Total protein (Input) or immunoprecipitation (IP) products using agarose conjugated with anti-Myc antibody (α-Myc) were probed by anti-Myc antibody, stripped, and re-probed by the anti-CRY1 antibody (α-CRY1). (G) Schematic representation depicting the domains of CRY1 and TCP2 that are required for CRY1–TCP2 interaction (shaded area). (This figure is available in colour at *JXB* online.)

A previous study showed that CIB1 interacted with CRY2 in a blue-light-dependent manner (Liu *et al.*, 2008a). Given the similar structure and functions of CRY1 and CRY2, we screened a yeast library (Pruneda-Paz *et al.*, 2014), which comprised >1000 Arabidopsis transcription factors, using a blue light-differentiated yeast two-hybrid assay to identify transcription factors that interact with CRY1. We identified a novel transcription factor, TCP2, which physically interacted with CRY1, and determined the specific wavelength of light required for the interaction (Fig. 1A). Yeast cells that co-expressed *TCP2* and *CRY1* exhibited appreciable β-gal activity in blue light, but no conspicuous β-gal activity was detected in red light or in darkness. This indicated that TCP2 interacted with CRY1 in a blue-light-dependent manner. CRY1 exhibited a stronger interaction with TCP2 in response to a higher blue light fluence rate (Fig. 1B). As shown in Fig. 1B, the intensity of TCP2–CRY1 interaction increased with an increase in the light fluence rate from 10 μmol m^–2^ s^–1^ to 40 μmol m^–2^ s^–1^. The stronger the blue light fluence rate, the faster was the increase in the intensity of their interaction (Fig. 1C).

We further examined the dependence of the CRY1–TCP2 interaction on blue light in plant cells using a BiFC assay. *Nicotiana benthamiana* leaves were co-transformed with two plasmids, which expressed the N-terminal region of yellow fluorescent protein (nYFP)–TCP2 or the C-terminal region of cyan fluorescent protein (cCFP)–CRY1 fusions, respectively. The co-transformed tobacco leaves were incubated in darkness or illuminated with blue light. We quantified the CRY1–TCP2 interaction by the percentages of cells with BiFC signals and found signals present in >30% of the cells in blue light and in 10% of the cells in darkness (Fig. 1D, E). This demonstrates that blue light enhanced the interaction between TCP2 and CRY1 in plant cells (*P*=0.003, Student’s *t*-test). The BiFC signals were detected primarily in the nucleus (Fig. 1D), which was consistent with the location of TCP2 and suggested that CRY1 and TCP2 function in the nucleus (Supplementary Fig. S1 at *JXB* online). GFP signals were detected in tobacco leaf cells, root tip cells of TCP2–GFP transgenic plants, and in the nucleus of TCP2–GFP transgenic seedlings. All the results, summarized in Supplementary Fig. S1, showed that TCP2 was a nuclear protein.

We used yeast two-hybrid and BiFC assays to map the interacting domains of CRY1 and TCP2. In addition to full-length TCP2 (TCPFL), we defined three TCP2 fragments which contained the TCP domain, TCPn (a TCP domain lacking the R domain), R&CT, and CT domains for use in the yeast two-hybrid assay based on published data (Cubas *et al.*, 1999; Martin-Trillo and Cubas, 2010). An overview of the CRY1–TCP2 domain interactions is shown in Fig. 1G. Compared with other domain interactions, the interaction between CRY1 and the TCPn domain exhibited the highest blue light specificity in the auxotrophy and liquid assays (Supplementary Fig. S2A, B at *JXB* online). Conversely, interaction between CRY1 and the TCP domain showed activity without blue light specificity. The interactions between CRY1 and the R&CT or CT domains were weaker than those with the fragments containing the TCP domain.

As previous studies indicated that CRY2N^489^ was required for CRY2 to interact with other proteins (Zuo *et al.*, 2011), we examined whether the corresponding CRY1 domain could interact with TCP2. However, due to the strong self-activation of CRY1N^493^, which was conserved in CRY2N^489^, we analyzed CRY1N^505^, CRY1N^515^, and other fragments of CRY1 for TCP2–CRY1 interaction. The auxotrophy assay and β-gal assay shown in Supplementary Fig. S3A, B at *JXB* online revealed that the CRY1 N-terminus (including CRY1N^505^, CRY1N^515^, and CRY1N^493^) interacted with TCP2. We confirmed that CRY1N^505^ and CRY1N^515^ interacted with TCP2 in a blue-ligh-dependent manner from Supplementary Fig. S3A and partly from Supplementary Fig. S3B, but we cannot confirm the interaction of CRY1N^493^ and TCP2 in the same manner due to the strong self-activation of CRY1N^493^ in both the auxotrophy assay and β-gal assay. Moreover, BiFC assays confirmed that CRY1N^505^ interacted with TCP2 in plant cells (Supplementary Fig. S2C, D). Taken together, these results suggested that TCP2 and CRY1 interacted via their N-terminal regions, that the TCP domain was important for the CRY1–TCP2 interaction, and that the R domain of TCP2 could affect the blue light specificity of CRY1–TCP2 interactions.

In addition, we examined whether CRY1 and TCP2 could form a complex in Arabidopsis. Transgenic plants overexpressing Myc-*TCP2* were exposed to blue light or kept in darkness and then subjected to co-immunoprecipitation assays using anti-CRY1 and anti-MYC antibodies (Fig. 1F). The results of this experiment demonstrated that *TCP2* formed a complex with CRY1 in Arabidopsis cells; however, no obvious blue light specificity was detected in this assay. Taken together, the results suggest that TCP2 physically interacts with CRY1 in yeast and plant cells.

### Light regulates the TCP2 protein expression level

**Fig. 2.**
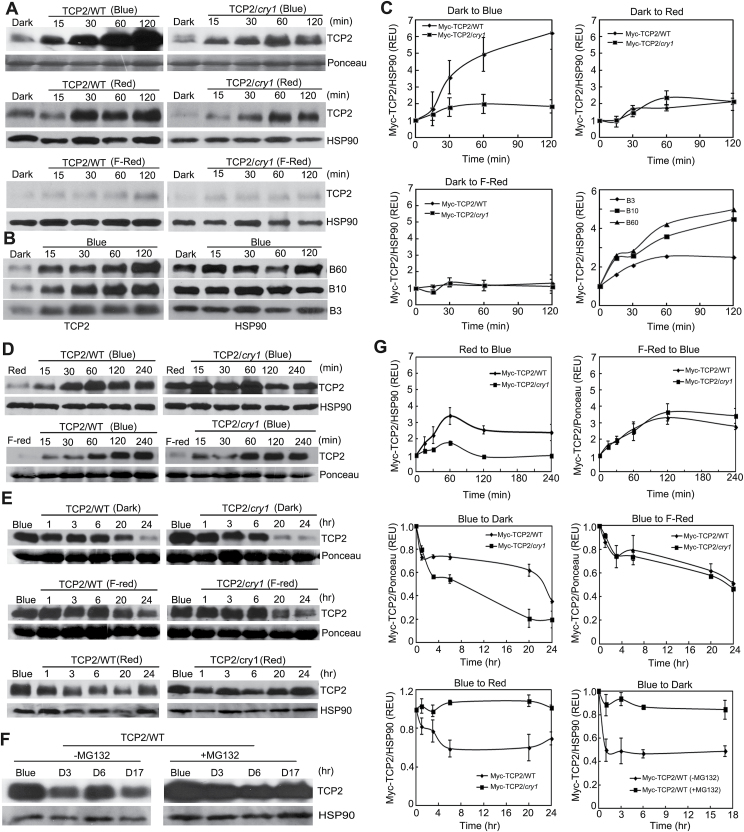
TCP2 is a light-regulated protein. (A) Immunoblots showing the effect of light on the expression of Myc-TCP2 protein in transgenic lines expressing 35S::*Myc-TCP2*. Plants were grown in long days (LDs; 16h/8h) for 3 weeks, moved to darkness for 16h, then exposed to 20 μmol m^−2^ s^−1^ far-red light, 18 μmol m^−2^ s^−1^ red light, or 20 μmol m^−2^ s^−1^ blue light for the indicated time. Samples were fractionated by SDS–PAGE, blotted to a nitrocellulose membrane, and probed with the anti-Myc antibody; HSP90 protein or the amounts of Rubisco (Ponceau) indicate relative loading of samples. (B) TCP2 protein accumulation increased as the blue light fluence rate increased. Plants grown in LDs (16h/8h) for 3 weeks were transferred to darkness for 16h, and then exposed to blue light of the indicated fluence rate (B 3, 3 μmol m^−2^ s^−1^, B 10, 10 μmol m^−2^ s^−1^, B 60, 60 μmol m^−2^ s^−1^) and time. Samples were analyzed by immunoblotting. (C) The immunoblots shown in (A and B) was quantified (Image J), calculated by the formula [(MYC-TCP2)^t^/Ponceau)^t^]/ [(Myc-TCP2)^0^/Ponceau^0^] or [(MYC-TCP2)^t^/(HSP90)^t^]/[(Myc-TCP2)^0^/HSP90^0^]; (Myc-TCP2)^0^ and (MYC-TCP2)^t^ are the signal at time zero and time indicated of Myc-TCP2, respectively; Ponceau^0^ and Ponceau^t^ are thesignal at time zero and time indicated of Rubisco protein, respectively; HSP90^0^ and HSP90^t^ are the signal at time zero and time indicated of HSP90, respectively. (D) TCP2 protein accumulated in blue light. Three-week-old *TCP2*/WT and *TCP2*/*cry1* transgenic plants grown in a LD (16h/8h) photoperiod were moved to red light (20 μmol m^−2^ s^−1^) and far-red light (20 μmol m^−2^ s^−1^) for 16h, and then transferred to blue light (35 μmol m^−2^ s^−1^) for the indicated time before sample collection. (E) Analysis of TCP2 protein expression level change when transferred from blue light to darkness, far-red light, and red light. Three-week-old LD- (16h/8h) grown plants were transferred to blue light (35 μmol m^−2^ s^−1^) for 16h, and then transferred to darkness, far-red light (20 μmol m^−2^ s^−1^), and red light (20 μmol m^−2^ s^−1^) for the indicated time before sample collection. (F) Analysis of TCP2 protein degradation mechanism. Plants were grown in white light in LDs (16h/8h) for 3 weeks, moved to blue light (35 μmol m^−2^ s^−1^) for 16h, and leaves were excised and incubated in the presence of MG132 solution (50 μmol l^–1^) or in its absence (0.1% DMSO) for 4h, after which they were transferred to darkness for the indicated time before sample collection. (G) The immunoblots shown in (D–F) were quantified (Image J). The formula used for calculation is the same as that in (C). (This figure is available in colour at *JXB* online.)

We investigated the effect of light on TCP2 protein expression levels to elucidate the role of TCP2 in light regulation during plant development. Transgenic Arabidopsis plants constitutively expressing epitope-tagged TCP2 (35S::*Myc-TCP2*) in WT and *cry1* mutant backgrounds were used to analyze the TCP2 protein expression level. In the first experiment, both types of transgenic plants were grown under an LD photoperiod (16h/8h) for 3 weeks and then transferred to darkness for 16h. After exposure to blue light, red light, or far-red light for a given time, the level of TCP2 protein was analyzed. The results showed that little TCP2 protein was detected in plants of either the WT background or *cry1* background pre-treated in darkness, but the level increased in blue light and red light, especially in the WT background (>6-fold) within 2h of the blue light treatment. No appreciable change of TCP2 protein expression level was detected when the plants were transferred from darkness to far-red light. The TCP2 protein level increased faster in the WT background than that in the *cry1* mutant background (Fig. 2A, C). The LUC–TCP2 fusion protein expression level changes in 6-day-old *LUC-TCP2*/WT, *LUC-TCP2*/*cry1*, *LUC-TCP2*/ *ztl3*, and *LUC-TCP2*/*cry1cry2* transgenic seedlings were similar to those shown in Fig. 2A when transferred from dark to blue light with various treatment times (Supplementary Fig. S4 at *JXB* online), corroborating the results of Fig. 2A.

In the second experiment, we investigated whether or not the TCP2 protein level responds to the photon dosage of blue light. Samples were pre-treated as described in Fig. 2A, then exposed to different light fluence rates of blue light (Fig. 2B, C). The results of this experiment demonstrated that the level of the TCP2 protein increased in response to higher fluence rates of blue light. Together, our results establish that the level of TCP2 protein expression is positively regulated by light in a wavelength-specific and photon density-dependent manner.

On the other hand, although previous results elucidated a red light or far-red light influence on the TCP2 protein level, blue light transcended the other lights to stimulate TCP2 expression. The TCP2 protein level was increased when the plants were transferred from red or far-red light to blue light, but not in the *cry1* background which showed no obvious change when transferred from red to blue light (Fig. 2D, G). Taken together, our results indicate that CRY1 mediates blue light stimulation of the TCP2 protein expression level. Besides the accumulation in blue light, TCP2 also became stable when transferred from darkness to red light (Fig. 2A); however, TCP2 protein exhibited a decrease from blue to red light in the WT background, suggesting that TCP2 accumulated predominantly in blue light (Fig. 2E, G).

TCP2 protein is stabilized by light but is labile in darkness; however, the relevant control mechanisms remain unknown. Given that ubiquitin/26S proteasome-dependent proteolysis is a common mechanism in the degradation of protein during light signal transduction (Lau and Deng, 2012), the TCP2 protein expression level was examined in the presence or absence of the 26S proteasome inhibitor, MG132, in TCP2/WT lines. Tissue samples were harvested and incubated in either the presence or the absence of MG132 in darkness for 17h and analyzed by immunoblotting. As expected, in the absence of MG132, TCP2 protein levels decreased markedly, but, when MG132 was present, TCP2 levels only decreased slightly (Fig. 2F, G), suggesting that the decrease of the TCP2 protein expression level in the absence of blue light is due to proteolysis of TCP2 by the 26S proteasome.

### TCP2 is a positive regulator of photomorphogenesis

**Fig. 3.**
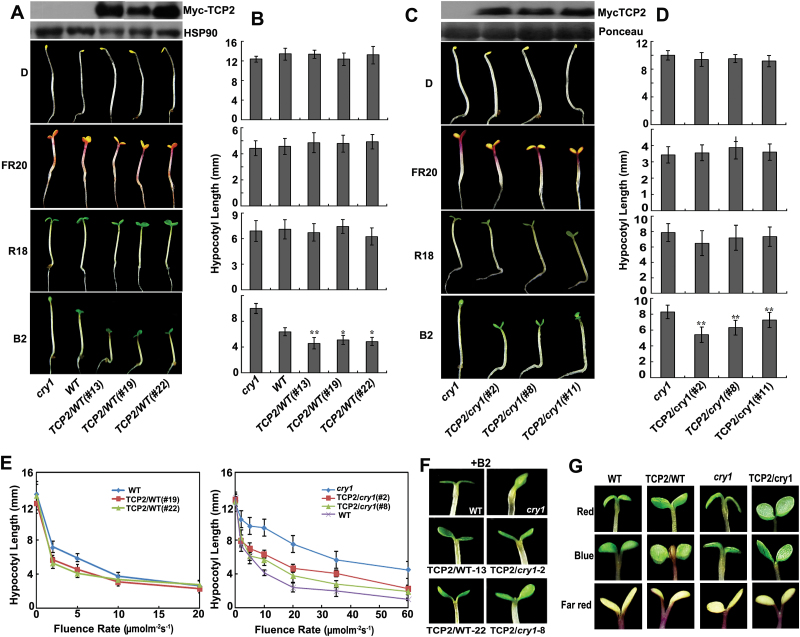
Light response phenotype analysis of transgenic lines with altered *TCP2* expression in different backgrounds. (A) Images of the hypocotyl phenotypes of 5-day-old *cry1*, WT, and *Myc-TCP2*/WT transgenic seedlings grown under different light (D, darkness; FR 20, far-red 20 μmol m^−2^ s^−1^; R 18, red light 18 μmol m^−2^ s^−1^; B 2, blue light 2 μmol m^−2^ s^−1^). Western blots show the overexpression of *TCP2* in the WT. Error bars represent ±SD of three biological replicates. (B) Hypocotyl lengths of the genotypes indicated in (A) grown in the different light conditions as in (A) were measured and are shown. The *P*-values of the hypocotyl length difference between WT and transgenic lines were 0.006, 0.027, and 0.014 for *TCP2/*WT (#13, # 19,#22) in B 2 (blue light 2 μmol m^−2^ s^−1^), respectively. (C) Images of hypocotyl phenotypes of 5-day-old *cry1* and *Myc-TCP2*/*cry1* transgenic seedlings grown under different light (D, darkness; FR 20, far-red light 20 μmol m^−2^ s^−1^; R 18, red light 18 μmol m^−2^ s^−1^; B 2, blue light 2 μmol m^−2^ s^−1^). Western blot shows the overexpression of *TCP2* in *cry1*. (D) Hypocotyl lengths of the genotypes indicated in (A) grown in the different light conditions as in (C) were measured and are shown. Error bars represent ±SD of three biological replicates. The *P*-values of the hypocotyl length difference between *cry1* mutant and transgenic lines were <0.0001, <0.0001, and 0.00032 for *TCP2*/*cry1* (#2, #8, #11) in B 2 (blue light 2 μmol m^−2^ s^−1^), respectively. (E) Hypocotyl lengths of the indicated genotypes grown in the dark or in continuous blue light with different fluence rate were measured and are shown. Error bars represent ±SD of three biological replicates (*n ≥*20). (F) Cotyledon opening images of the seedlings show *TCP2* overexpression lines with the indicated genotypes and light treatment.(G) A rolled -upward cotyledon margin phenotype of *TCP2* overexpression lines as indicated appears in blue and red light, but not in far-red light. The light fluence rate was 10 μmol m^−2^ s^−1^. (This figure is available in colour at *JXB* online.)

To understand further the functions of the CRY1–TCP2 interaction in the CRY1 signal pathway, we examined the hypocotyl elongation of transgenic plants expressing the 35S*::Myc-TCP2* transgene. The *Myc-TCP2*/WT exhibited shorter hypocotyls than the WT in blue light, but no difference existed between these two in dark, far-red, or red light conditions (Fig. 3A, B). In addition, the mutants *tcp2*, *tcp2tcp4*, and *tcp2tcp4tcp10*, and the RNAi transgenic lines of TCP2 all exhibited slightly longer hypocotyls than their own backgrounds; the additional knockout of *tcp4*, or both *tcp4* and *tcp10*, in the *tcp2* background showed no effect additive to the elongated hypocotyls phenotype of the *tcp2* single mutant, suggesting that *TCP2* is the major member in the TCP family involved in regulation of hypocotyl elongation (Supplementary Fig. S5 at *JXB* online).

Next, the ability of *Myc-TCP2* to inhibit hypocotyl elongation in the *cry1* background was analyzed. Overexpression of *Myc-TCP2* partially rescued the *cry1* mutant phenotype after blue light irradiation (Fig. 3C, D). To investigate further, we analyzed the hypocotyl phenotype of *LUC-TCP2*-overexpressing transgenic lines in WT, *cry1*, and *cry1cry2* backgrounds. The hypocotyls of *LUC-TCP2*/WT and *LUC-TCP2/cry1* seedlings were moderately shorter than those of WT and *cry1*, respectively, in blue light; however, hypocotyl lengths were only marginally affected by blue light when *LUC-TCP2* was expressed in the *cry1cry2* background (Supplementary Fig. S6 at *JXB* online). This suggests that both *CRY1* and *CRY2* partially mediate blue light inhibition of hypocotyl elongation associated with TCP2, which is consistent with the overlapping roles of *CRY1* and *CRY2* in regulation of the TCP2 protein expression level under blue light (Supplementary Fig. S4). In addition, TCP2 inhibited hypocotyl elongation in a manner dependent on the photon dosage of blue light (Fig. 3E). *Myc-TCP2*/WT seedlings were shorter than WT seedlings under blue light with relatively low fluence rates (<5 μmol m^−2^ s^−1^) (Fig. 3E), but the difference between these two decreased under blue light with higher fluence rates (>10 μmol m^−2^ s^−1^) and disappeared at the highest fluence rate tested (20 μmol m^−2^ s^−1^) (Fig. 3E). A similar but more pronounced difference occurred in *Myc-TCP2*/*cry1* and *cry1*, and this difference was maintained at fluence rates up to 60 μmol m^–2^ s^–1^ (Fig. 3E). These results suggest that TCP2 positively regulates hypocotyl inhibition imposed by blue light.

TCP2 inhibition of hypocotyl elongation was further verified by cotyledon expansion; *Myc-TCP2*/*cry1* and *Myc-TCP2*/WT seedlings presented an early cotyledon opening phenotype (Fig. 3F). Interestingly, the cotyledon margins of *TCP2* overexpression lines in WT and *cry1* mutant backgrounds rolled upward both in blue light and in red light, but not in far-red light (Fig. 3G), suggesting that TCP2 acts on the hypocotyl and cotyledon in different signaling pathways.

### TCP2 mediates light regulation of gene expression

**Fig. 4.**
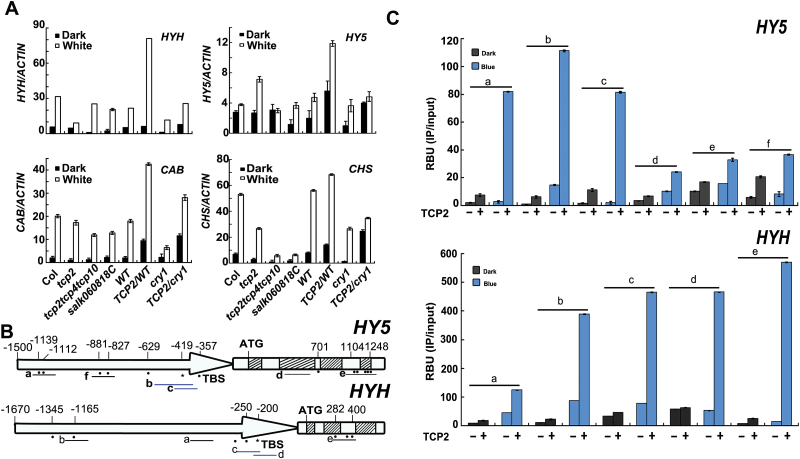
*TCP2* regulates the expression of genes downstream of the *CRY1* signaling pathway and binds to the chromatins of *HY5* and *HYH* to promote photomorphogensis. (A) mRNA expression of genes downstream of *CRY1* in different *TCP2* genotypes as indicated. Seedlings were grown in continuous white light for 10 d, and moved to darkness or remained in continuous white light for 33h before tissues were collected. (B) Diagrams depicting the promoter (arrow) and genome (white box, intron; striped box, exon) of *HY5* and *HYH*. The black circles and asterisk indicate the position of potential TCP-binding sites (the asterisk indicates the position of published TCP Class II gene-binding sites; circles indicate the positions of sequences which are partly similar to the published binding sites). Different regions of the *HY5* and *HYH* genomic DNA examined by ChIP-qPCR are indicated with short underlines (underlining of the b and c regions of HY5, and the c and d regions of HYH mark the DNA regions including sequences indicated by an asterisk, and other underlines mark DNA regions including sequences indicated only by circles). (C) ChIP-qPCR analysis of the indicated chromatin regions of *HY5* and *HYH* in darkness and blue light (35 μmol m^−2^ s^−1^) of samples collected from the *TCP2*-overexpressing transgenic line and the *cry1* mutant background. Seedlings were grown in continuous white light for 10 d and then transferred to blue light or darkness for 2 d before sample collection. ChIP samples were prepared by the anti-Myc antibody and subjected to qPCR analysis. Results of ChIP-qPCR were quantified by normalization of the IP signal with the corresponding input signal. The SDs are shown (*n*=3). RBU (relative binding unit)=PCR signal of the IP reaction/PCR signal of the mock reaction without antibody. (This figure is available in colour at *JXB* online.)

To investigate how TCP2 mediates light-regulated inhibition of hypocotyl elongation, we examined the transcriptional expression of several genes involved in photomorphogenesis in various *TCP2* genotype backgrounds. As shown in Fig. 4A, the transcriptional expression of *CHS*, *CAB*, *HY5*, and *HYH* increased in *TCP2* overexpression lines, but decreased moderately in *tcp2* or *tcp2tcp4tcp10* mutants in light, except for *HY5* whose transcription expression level increased in the *tcp2* mutant. In Arabidopsis, the *HY5* and *HYH* genes encode positive regulators of photomorphogenesis, which overlap functionally with hypocotyl elongation and other aspects of plant development (Oyama *et al.*, 1997; Holm *et al.*, 2002). Therefore we investigated whether TCP2 interacted or not with *HY5* or *HYH* chromatin in dark and light conditions using *Myc-TCP2/cry1* and *cry1* seedlings. TCP-binding sites (TBSs; GTGGNCCC/TGGGCC) and potential binding sites were identified in the *HY5* and *HYH* chromatin regions, which extended from –1500bp and –1670bp to the start codon, respectively, based on the binding sites of TCP Class II reported in a previous study (Kosugi and Ohashi, 2002; Danisman *et al.*, 2012) (Fig. 4B). We defined six *HY5* chromatin regions (a–f) and performed ChIP-PCR experiments to screen regions bound by TCP2. TCP2 protein bound to *HY5* chromatin regions a–c but had no obvious interaction with regions d–f Supplementary Fig. S7 at *JXB* online). Subsequently, ChIP-qPCR was employed to confirm the screening results. The results in Fig. 4C showed that TCP2 protein had a higher affinity for *HY5* chromatin regions a–c than for regions d–f in blue light. ChIP-qPCR was also performed to examine the interactions of *HYH* chromatin regions with TCP2 protein. The results suggested that an apparent binding activity difference occurred between regions b–e and a; regions b–e had an obviously higher affinity than region a for TCP2 protein in blue light. In addition, TCP2 protein showed low or even no affinity for both *HY5* and *HYH* chromatins in darkness when compared with the interactions in blue light (Fig. 4C). Notably the regions without TBSs or potential similar binding sites exhibited weak affinity for TCP2 protein. Finally, our results suggest that TCP2 interacts with the *HY5* and *HYH* chromatins to regulate their transcription and subsequent hypocotyl elongation.

To understand further how TCP2 mediates light-regulated plant photomorphogenesis, we examined endogenous *TCP2* mRNA expression in various light receptor mutants. Our result indicated that *TCP2* was down-regulated in various light receptor mutants, but up-regulated in the *cop1-4* mutant under blue light (Supplementary Fig. S8A, B at *JXB* online). In addition, blue light fluence rates interacted with *CRY1* to increase *TCP2* mRNA levels (Supplementary Fig. S8C). These results suggest that light regulates the expression of TCP2 at both the transcriptional and post-transcriptional levels. To test the *TCP2*-related genotypes, we used the lipoxygenase 2 gene (*LOX2*) as a marker. *LOX2* is an approved target gene of TCP proteins, and its product, LOX2, is a key enzyme that regulates the synthesis of jasmonic acid (Schommer *et al.*, 2008; Danisman *et al.*, 2012). Corroborating previous studies, *LOX2* and *TCP2* expression levels were positively correlated in WT, *cry1*, and *TCP2*-related genotypes (Supplementary Fig. S9 at *JXB* online). Furthermore, *LOX2* expression was similar to that of *TCP2* in *hy5hyh* and *cop1-6* mutants. In summary, our results suggest that *TCP2* positively regulates *CHS*, *CAB*, *HY5*, and *HYH*. Conversely, transcriptional expression of *TCP2* was lower in *hy5hyh* mutants than in the WT (Supplementary Fig, S10), suggesting possible feedback regulation among *TCP2*, *HY5*, and *HYH* under light.

## Discussion

TCP2 belongs to the Class II subfamily of the TCP family and has conserved TCP and R domains. The high identity and similarity of multiple Class II genes in the genome of Arabidopsis suggest that they may share redundant functions in plant development (Martin-Trillo and Cubas, 2010).

In previous studies, several proteins have been reported to interact physically or genetically with CRY1, including SPA1 (Liu *et al.*, 2011), PHYA (Ahmad *et al.*, 1998b), and COP1 (Wang *et al.*, 2001; Yang *et al.*, 2001). However, whether *CRY1* directly regulates transcription remains unclear. In this study, we identified TCP2 as a novel transcription factor protein that interacted with CRY1 in a blue-light-dependent manner in yeast and tobacco leaf cells (Fig. 1A–E); however, no obvious blue light specificity was detected in Arabidopsis cells (Fig. 1F). Based on the detectable blue light specificity of the TCP2–CRY1 interaction in yeast and tobacco systems, which are systems free of Arabidopsis proteins, it was hypothesized that another Arabidopsis protein was involved in the TCP2–CRY1 complex to anchor the TCP2 in darkness and facilitate the CRY1–TCP2 signal transduction in blue light. Another option is technical and due to the nature of the implemented transient technologies with high expression levels. The TCP domain is involved in protein–protein interactions by virtue of its formation of homo- or heterodimers with other proteins (Cubas *et al.*, 1999; Kosugi and Ohashi, 2002). In addition, the requirement for the photolyase-related (PHR) domain for the interaction of CRY1 (Sang *et al.*, 2005) indicates that the interaction between CRY1 and TCP2 may be due to interaction of their N-terminal regions.

In this study, TCP2 protein levels increased markedly under blue light, and to a slightly lesser degree under red light, but TCP2 was degraded by the 26S proteasome in darkness (Fig. 2). Furthermore, the TCP2 protein level was affected by red light, suggesting an involvement of phytochrome in the regulation of the TCP2 protein expression level. Another fact is that blue light transcends red light or far-red light to affect TCP2 protein accumulation or degradation (Fig. 2D, E, G). CRY1 mediated blue light regulation of TCP2 expression through both post-transcriptional and post-translational mechanisms (Fig. 2; Supplementary Figs S4, S8C at *JXB* online). We demonstrated that *TCP2* can be regulated by different light receptors (see Supplementary Figs S4, S8A, B), but further investigation is needed to determine how TCP2 expression is regulated by them. Moreover, we found that CRY1 and CRY2 functioned redundantly to regulate TCP2 expression and associate with TCP2 function in hypocotyl elongation (see Supplementary Figs S4, S6, S8). Overexpression of *TCP2* inhibited the hypocotyl elongation in blue light and affected cotyledon morphology in blue light or red light, but not in far-red light, indicating that different signal pathways mediated by TCP2 are involved in hypocotyl and cotyledon developments (Fig. 3).

We found that TCP2 affected the mRNA expression of downstream genes of the CRY1 signaling pathway such as *CAB*, *CHS*, *HY5*, and *HYH*. Possible explanations for the different expression change of *HY5* in the *tcp2* mutant in light may include the competitive regulation of TCP Class II genes. TCP Class II genes showed similar binding preferences, causing the competition (Kosugi and Ohashi, 2002). The mutation of *TCP2* provides more opportunities for other TCP Class II genes such as *TCP4* and *TCP10* to bind *HY5* mRNA and regulate its expression. Their positive regulation of *HY5* mRNA expression complements its down-regulation resulting from the mutation of *TCP2* and even surpasses *TCP2* to promote *HY5* mRNA expression in light. Another possible reason is the existence of an as yet unidentified regulation pathway or protein–protein interaction (Giraud *et al.*, 2010). In addition, blue light affected the molecular interactions between TCP2 protein and the chromatins of *HY5* or *HYH* (Fig. 4), suggesting that TCP2 may regulate hypocotyl elongation through *HY5* or *HYH*. However, several factors remain to be investigated: what is the real reason causing the change in *HY5* transcription in the *tcp2* mutant; what is the reason for the increased binding of chromatin regions of *HY5* and *HYH* to TCP2 protein in blue light, whether it is due to the amount of TCP2 protein promoted by blue light as shown in Fig. 2 or to the activity of TCP2 protein stimulated by blue light need further investigation; how many target genes of TCP2 are in the CRY1 signal pathway; which protein is involved in the CRY1–TCP2 interaction; and how do other light receptors regulate TCP2 to mediate plant development. Therefore, additional studies are needed to elucidate the questions posed above.

## Supplementary data

Supplementary data are available at *JXB* online.

Figure S1. TCP2 is a nuclear protein.

Figure S2. Analyses of interaction domains of CRY1 and TCP2.

Figure S3. Analyses of the interaction of the CRY1 domains and TCP2 in yeast cells.

Figure S4. Luciferase assay showing the accumulation of LUC–TCP2 in different backgrounds.

Figure S5. Hypocotyl phenotypes of TCP2 mutants with response to light.

Figure S6. Light response hypocotyl phenotype analysis of LUC–TCP2 in WT, *cry1*, and *cry1cry2* backgrounds.

Figure S7. ChIP-PCR for screening of TCP2-interacting *HY5* chromatin regions.

Figure S8. *TCP2* mRNA expression in response to light and regulation by different light photoreceptors.

Figure S9. TCP2 target gene *LOX2* mRNA expression in different genotypes of *TCP2* as indicated.

Figure S10. *TCP2* and its downstream gene *LOX2* mRNA expression in *hy5hyh* and *cop1-6* mutants.

Table S1. Oligonucleotide primers used in this study.

Supplementary Data

## Abbreviations:

CIB1: CRYPTOCHROME-INTERACTING BASIC–HELIX–LOOP HELIX1;
CO: CONSTANS;
COP1: CONSTITUTIVELY PHOTOMORPHOGENIC1
CRY1: CRYPTOCHROME 1
CRY2: CRYPTOCHROME 2
HFR1: REDUCED SENSITIVITY TO FAR-RED LIGHT 1
HY5: ELONGATED HYPOCOTYL 5
HYH: HY5-HOMOLOG
SPA1: SUPPRESSOR OF PHYTOCHROME A1
TCP2: TEOSINTE-LIKE1, CYCLOIDEA, AND PROLIFERATING CELL FACTOR 2.
