# Supplementary material for: TCP2 positively regulates HY5/HYH and photomorphogenesis in Arabidopsis
Source: J Exp Bot. 2015 Nov 23;67(3):775–85. doi: 10.1093/jxb/erv495 (PMC4737077; doi:10.1093/jxb/erv495)
Supplement: Supplementary Data [file supp_67_3_775__index.html]

TCP2 positively regulates HY5/HYH and photomorphogenesis in Arabidopsis — TCP2 positively regulates HY5/HYH and photomorphogenesis in Arabidopsis — Supplementary Data 

# TCP2 positively regulates *HY5/HYH* and photomorphogenesis in Arabidopsis

## Supplementary Data

Data files

- Supplementary\_Figure\_S1\_S10\_Table\_S1.pdf - Supplementary Data
