# Supplementary material for: TCP2 positively regulates HY5/HYH and photomorphogenesis in Arabidopsis
Source: J Exp Bot. 2015 Nov 23;67(3):775–85. doi: 10.1093/jxb/erv495 (PMC4737077; doi:10.1093/jxb/erv495)
Supplement: Supplementary Data [file supp_erv495_Supplementary_Figure_S1_S10_Table_S1.pdf]

Supplemental data

SUPPLEMENTAL FIGURE LEGENDS

Figure S1. TCP2 is a nuclear protein.

(A-C) Fluorescent microscopy images of the *35S:: TCP2-GFP* expressed in tobacco leaves.(A) DAPI (4',6-diamidino-2-phenylindole) stain showing nuclei.(B) images of GFP, showing GFP signal in nucleus.(C) Merge of (A) and (B);scale bar= 10 μm

(D-F) Fluorescent microscopy images of the *35S:: TCP2-GFP* expressed in Arabidopsis transgenic lines root tips. D-F images the same to A-C respectively; scale bar= 10 μm

(G-I) Nucleus fluorescent microscopy images of *35S:: TCP2-GFP*. 7-day-old continuous white light grown transgenic lines expressed *35S:: TCP2-GFP* was sampled to extract the nucleus. G, H, I image the same to A, B, C respectively; scale bar= 2 μm

Figure S2. Analyses of domain interactions of CRY1 and TCP2.

(A) An auxotrophy growth assay of yeast two-hybrid experiment showing the interactions of CRY1 and TCP2 domains. For Histidine auxotrophy assay, the yeast cells plated on the SD/-Leu/-Trp/-His/-ade with 10 mM 3-AT (top), SD/-Leu/-Trp (bottom) in the blue light 35 μmol m^{-2} se^{c-1} and darkness for three days.

(B) β-gal assay showing the interactions of CRY1 with TCP2 domains in the yeast cells in Darkness or Blue (blue light, 35 μmol m^{-2} sec^{-1})

(C) BiFC assay showing the interaction of CRY1 N^{505} domain and TCP2 in *N. benthamiana* leaf cells. Four weeks *N. benthamiana* leaves infiltrated with the *Agro bacteria* mix which harbored the plasmids as indicated. Leaves were infiltrated and then incubated in dark for 12 hr, moved to white light for 48 hr until fluorescence microscope assay. DAPI, nucleus fluorescence; YFP, YFP fluorescence; Merge, Merge of DAPI and YFP. Scale bar=10 μm

(D) The percentage of *N. benthamiana* leaf cells that showed the BiFC fluorescence signals in (C) were counted. Each samples contained at least 150 cells. Means and standard deviations (n=3) are shown, *P*=0.001, 0.007 (Student's *t* test).

Figure S3. Analyses of the CRY1 domain and TCP2 interaction in yeast cells.

(A) The auxotrophy assay showing the domains of CRY1 required for the CRY1-TCP2 interaction. Left: constructs of CRY1 domains used for analysis. Right: Histidine auxotrophy of yeast two-hybrid experiments showing the blue light-specificity interaction of CRY1N ^{505} or CRY1N^{515} with TCP2. The yeast cells (AH109) were placed on the SD/-Leu/-Trp/-His/-ade with 10 mM 3-AT medium and cultured in blue light (B, 35 μmol m^{-2} sec^{-1}), Red light (R, 18 μmol m^{-2} sec^{-1}) ,far red light (FR, 20 μmol m^{-2} sec^{-1}) or darkness (D) for 3 days.

(B) β-galactosidase activities (Miller units) determining the domains of CRY1 required for the CRY1-TCP2 interaction. Yeast cells were exposed to blue light or remained in darkness for 4 hr. The *p* value of the interactions of TCP2 with CRY1, CRY1N^{505}, CRY1N^{493} were < 0.0001 both in blue light and darkness; The *p* values of TCP2 interaction with CRY1N^{515} were 0.024 and 0.087 in blue light and darkness, respectively.

Figure S4. Luciferase assay showing the accumulation of *LUC-TCP2* in different backgrounds

Transgenic line expressing the 35S::*LUC-TCP2* transgene in the WT (*LUC-TCP2*/WT) , cry1(*LUC-TCP2*/*cry1*), *cry1cry2* (*LUC-TCP2*/*cry1cry2*) and *ztl3* (*LUC-TCP2*/*ztl3*) mutant backgrounds were sown into 96 well plates and grown in continuous white light for 5 days ,transferred to darkness for 16 hr, and then exposed to blue light (35 μmol m^{-2} sec^{-1}) for indicated time, LUC signal was sensed by luminometer. The relative expression unit (REU) of LUC-TCP2 was calculated by the formula [(LUC-TCP2)^{t}/ (LUC-TCP2)^{0}], (LUC-TCP2)^{0} and (LUC-TCP2)$^{t:}$ luciferase signal at time zero and time indicated, respectively.

Figure S5. Hypocotyl phenotype of *TCP2* mutants with response to light.

(Left) Images of 5-old-days Col, *cry1*, WT, *tcp2*, *tcp2tcp4*, *tcp2tcp4tcp10* or

TCP2RNAi transgenic lines seedlings grown under different light (D, Darkness; FR 20, Far red 20 μmol m^{-2} sec^{-1}; B 2, Blue light 2 μmol $m^{-2}$$sec^{-1}$, B 10, Blue light 10 μmol m^{-2} sec^{-1}, B 15, Blue light 15 μmol m^{-2} sec^{-1}).

(Right) Hypocotyls lengths of the indicated genotypes in (Left) grown in different light conditions referred in (Left) were measured and shown. Error bars represent SD of three biological replicates. The *p* values of *TCP2RNAi-1* were 0.02 and 0.04 compared with the WT in B2 and B15, respectively, others as indicated in figure with the *p* value< 0.0001.

Figure S6. Light response hypocotyl phenotype analysis of LUC-TCP2 in WT, *cry1* and *cry1cry2* backgrounds.

(Left) Images of 5-old-days transgenic seedlings expressing *LUC-TCP2* in WT, *cry1* and *cry1cry2* backgrounds. Seedlings were grown under different light (D, Dark; FR 20, Far red 20 μmol m^{-2} sec^{-1}; R 20, Red light 20 μmol m^{-2} sec^{-1}; B 20, Blue light 20 μmol m^{-2} sec^{-1}) hypocotyl phenotypes. (Right) Hypocotyls lengths of the indicated genotypes in (left) grown in different light conditions referred in (left) were measured and shown. The *P*-value of the hypocotyls length difference between backgrounds (wild type and *cry1* mutant) and transgenic lines are < 0.001, whereas *p*=0.957 and 0.653 between *cry1cry2* and transgenic lines (#24 and #25) in B 20 (blue light 20 μmol m^{-2} sec^{-1}) respectively.

Figure S7. ChIP-PCR for TCP2 interacting *HY5* chromatin regions screening.

The lower cases (a, b, c, d, e, f) indicate the DNA regions of HY5 genomic DNA marked by short underlines in Fig. 4B. Samples for ChIP-PCR screening analysis were collected from ten days Myc-*TCP2/cry1* transgenic line and *cry1* mutant seedlings. ChIP samples were prepared by the anti-Myc antibody, absence of anti-Myc and Input (total DNA), and then subjected to PCR analysis.

Figure S8. *TCP2* mRNA expression in response to light and regulated by different light photoreceptors

(**A**) *TCP2* mRNA expression in indicated light receptor mutants. Seedlings grown in

continuous white light for 10 days before tissues collected.

(B) Time course expression of *TCP2* mRNA in WT, *cry1*, *cop1-4*, *cry1cry2*, *ztl3lkp2* mutants in response to blue light. Seedlings were grown in continuous white light for 10 days, moved to darkness for 16 hr, and then transferred to blue light 35 μmol m^{-2} sec^{-1} (B 35) for indicated time before tissues collected.

(C) *TCP2* mRNA expression in different blue light fluence rate in WT and *cry1* mutant. Seedlings grown condition was the same to (A), except that seedlings treated in different blue light fluence rate as indicated for 4 hr before tissues collected.

Figure S9. TCP2 target gene *LOX2* mRNA expression in different genotypes of *TCP2* as indicated.

Figure S10. *TCP2* and its downstream gene *LOX2* mRNA expression in *hy5hyh* and *cop1-6* mutants. Seeding grown condition and treatment before collected were the same to (Fig. 4A)

SUPPLEMENTAL FIGURES

Figure S1.

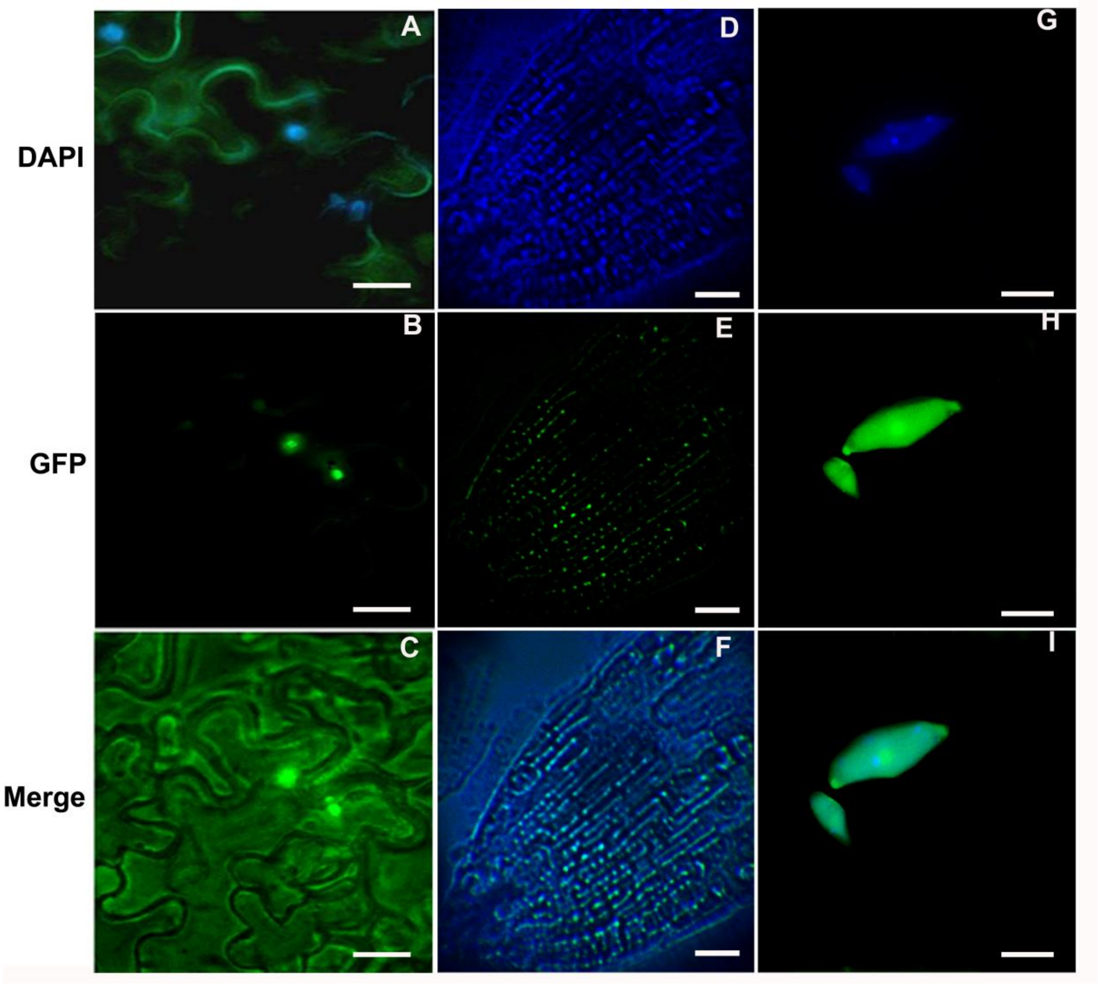

Figure S2.

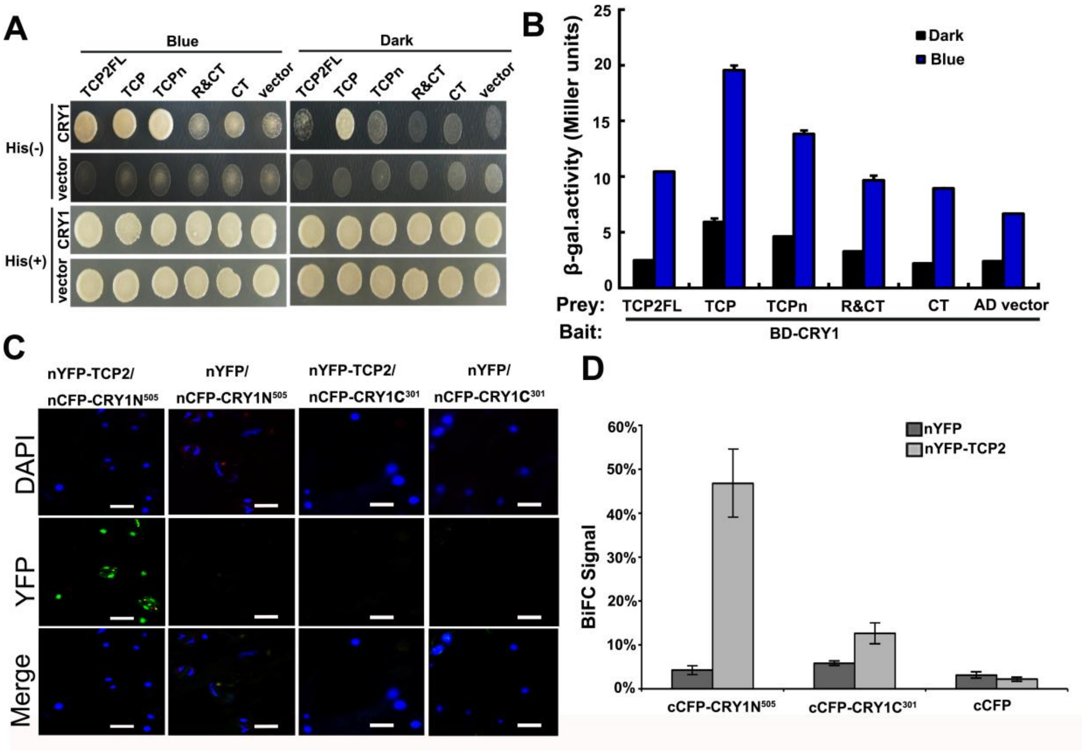

A
Blue
Dark
TCP2FL
TCP
TCPn
R&CT
CT
vector
His(-)
His(+)
CRY1
vector
B
β-gal.activity (Miller units)
Dark
Blue
Prey:
Bait:
AD vector
BD-CRY1
C
nYFP-TCP2/
nCFP-CRY1N505
nYFP/
nCFP-CRY1C301
DAPI
YFP
Merge
D
BiFC Signal
nYFP
nYFP-TCP2
cCFP-CRY1N505
cCFP-CRY1C301
cCFP

Figure S3.

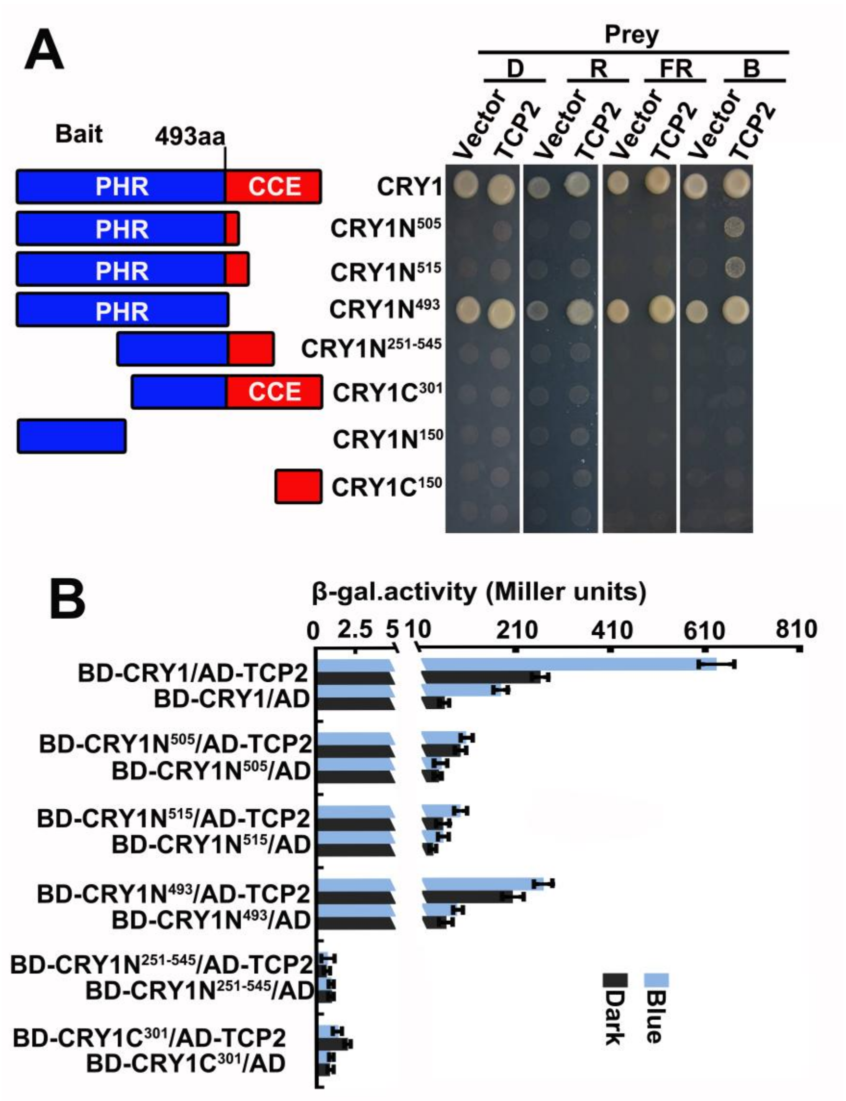

A
Prey
D
R
FR
B
Vector
TCP2
Bait
493aa
PHR
CCE
CRY1
CRY1N505
CRY1N515
CRY1N493
CRY1N251-545
CRY1C301
CRY1N150
CRY1C150
B
β-gal.activity (Miller units)
0
2.5
5
10
210
410
610
810
BD-CRY1/AD-TCP2
BD-CRY1/AD
BD-CRY1N505/AD-TCP2
BD-CRY1N505/AD
BD-CRY1N515/AD-TCP2
BD-CRY1N515/AD
BD-CRY1N493/AD-TCP2
BD-CRY1N493/AD
BD-CRY1N251-545/AD-TCP2
BD-CRY1N251-545/AD
BD-CRY1C301/AD-TCP2
BD-CRY1C301/AD
Dark
Blue

Figure S4.

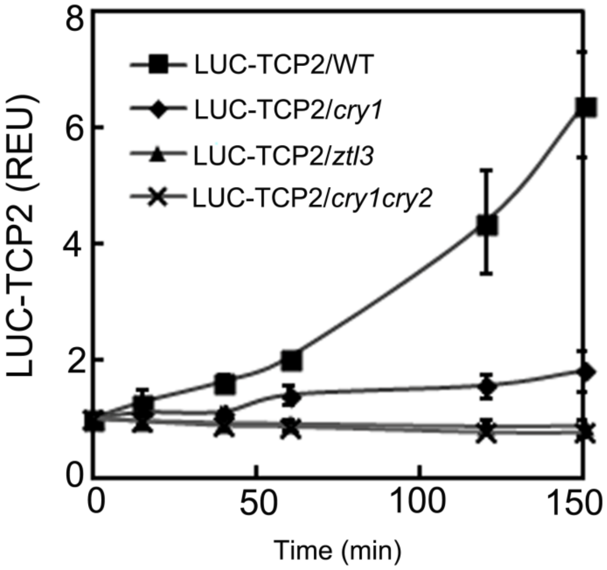
8
6
4
2
0
LUC-TCP2 (REU)
LUC-TCP2/WT
LUC-TCP2/*cry1*
LUC-TCP2/*ztl3*
LUC-TCP2/*cry1cry2*
0
50
100
150
Time (min)

Figure S5.

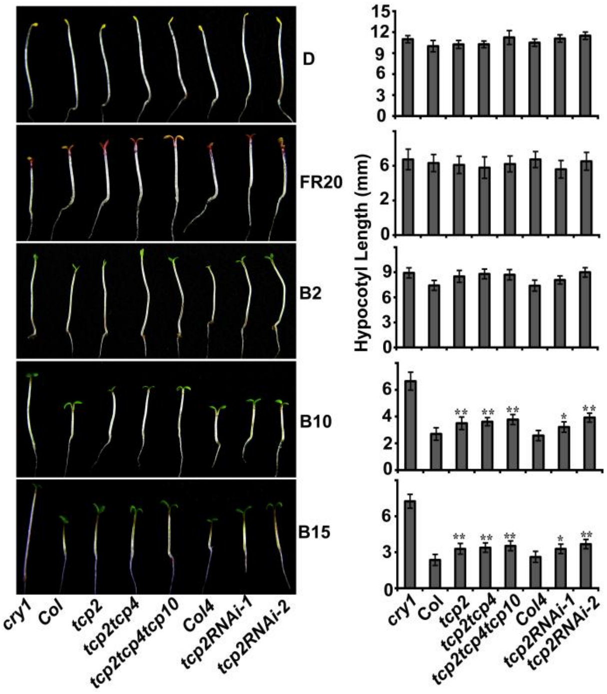

D
FR20
B2
B10
B15
cry1
Col
tcp2
tcp2tcp4
tcp2tcp4tcp10
Col4
tcp2RNAi-1
tcp2RNAi-2
Hypocotyl Length (mm)

Figure S6.

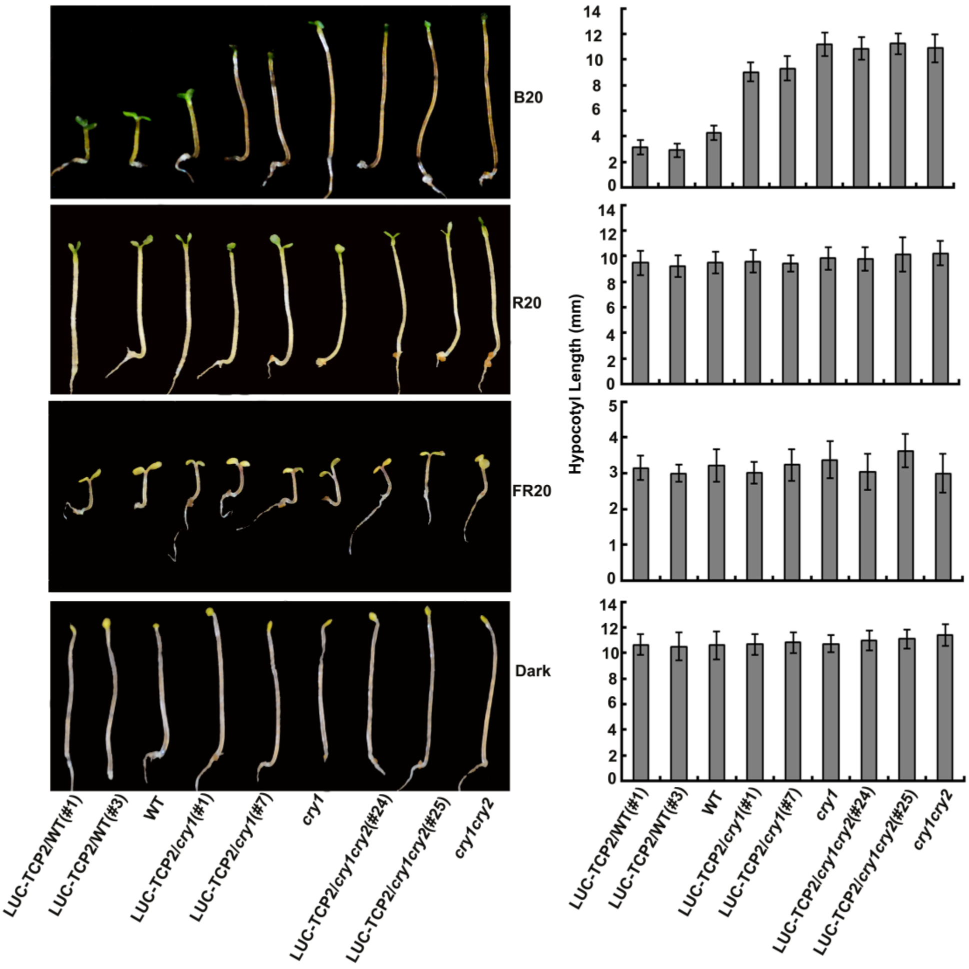
B20
R20
FR20
Dark
LUC-TCP2/WT(#1)
LUC-TCP2/WT(#3)
WT
LUC-TCP2/cry1(#1)
LUC-TCP2/cry1(#7)
cry1
LUC-TCP2/cry1cry2(#24)
LUC-TCP2/cry1cry2(#25)
cry1cry2
Hypocotyl Length (mm)
0
2
4
6
8
10
12
14
1
3
5

Figure S7.

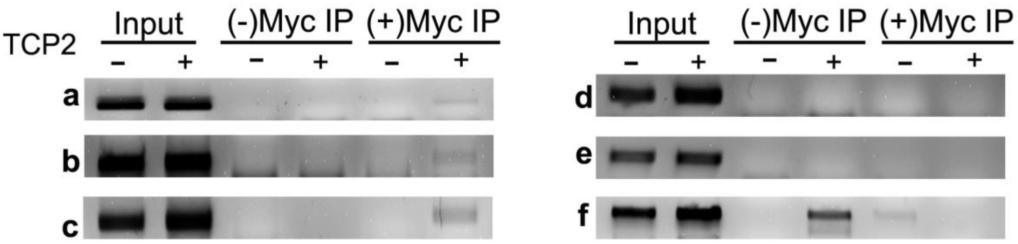

Figure S8.

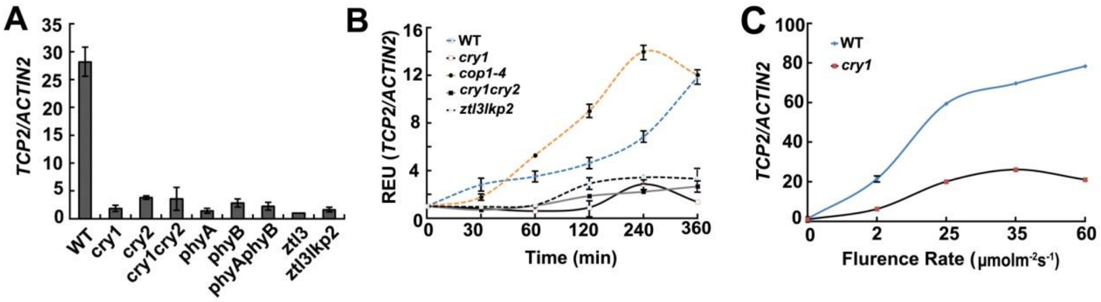

A
TCP2/ACTIN2
35
30
25
20
15
10
5
0
WT
cry1
cry2
cry1cry2
phyA
phyB
phyAphyB
ztl3
ztl3lkp2
B
REU (TCP2/ACTIN2)
16
12
8
4
0
WT
cry1
cop1-4
cry1cry2
ztl3lkp2
0
30
60
120
240
360
Time (min)
C
TCP2/ACTIN2
100
80
60
40
20
0
WT
cry1
0
2
25
35
60
Flurence Rate (μmolm-2s-1)

Figure S9.

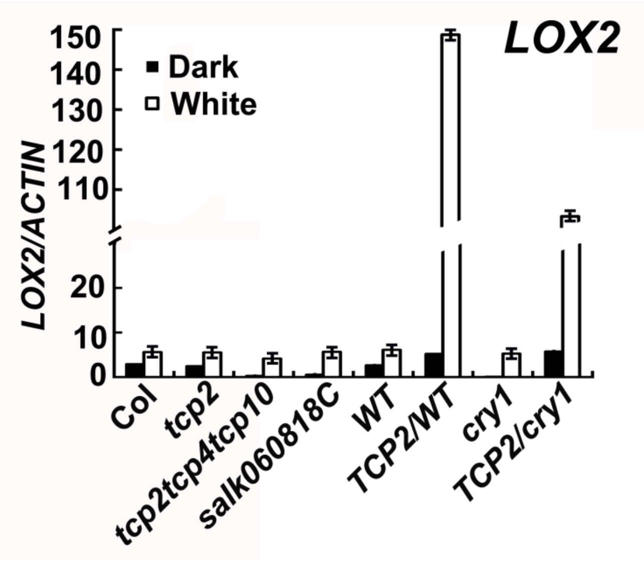
LOX2
Dark
White
LOX2/ACTIN
150
140
130
120
110
20
10
0
Col
tcp2
tcp2tcp4tcp10
salk060818C
WT
TCP2/WT
cry1
TCP2/cry1

Figure S10.

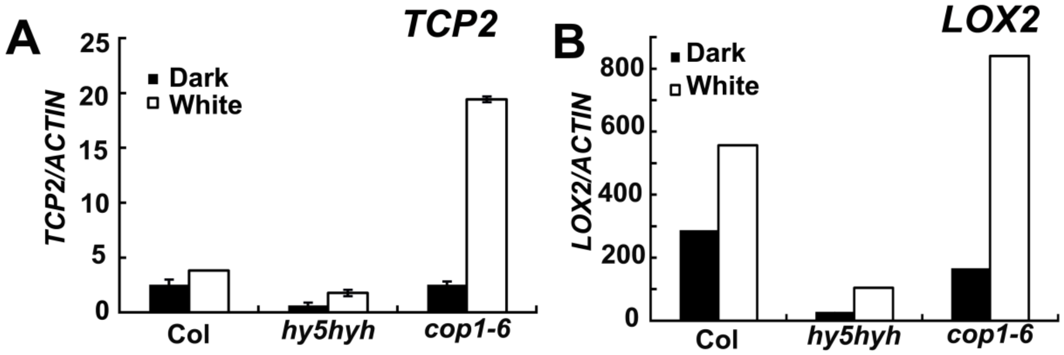

A
TCP2
TCP2/ACTIN
25
20
15
10
5
0
Dark
White
Col
hy5hyh
cop1-6
B
LOX2
LOX2/ACTIN
800
600
400
200
0
Dark
White
Col
hy5hyh
cop1-6

Table S1: Oligonucleotide primers used in this study

Gene cloning	TCP2-F	ATGATTGGAGATCTAATGAAGAATAAC
	TCP2-R	GTTCTTGCCTTTACCCTTATGTT
	CRY1FL-F	ATGTCTGGTTCTGTATCTGGTTGTG
	CRY1FL-R	CCCGGTTTGTGAAAGCC
	CRY1N^{505}-F	ATGTCTGGTTCTGTATCTGGTTGTG
	CRY1N^{505}-R	TCCGTTCTCTATTGCAGCTCTT
	CRY1N^{515}-F	ATGTCTGGTTCTGTATCTGGTTGTG
	CRY1N^{515}-R	CTCAGCAGAATCTCCAAGTCCTT
	CRY1N^{493}-F	ATGTCTGGTTCTGTATCTGGTTG
	CRY1N^{493}-R	TTGCCACATCTGTGAAAGC
	CRY1N$^{251\text{-}545}$-F	TCTCCACACTTGCATTTTGG
	CRY1N$^{251\text{-}545}$-R	ATCCTCATATCTCCTGTTTGGG
	CRY1C^{301}-F	AGGTACATAAGTTTTAACCATCCAT
	CRY1C^{301}-R	CCCGGTTTGTGAAAGCC
	CRY1N^{150}-F	ATGTCTGGTTCTGTATCTGGTTGT
	CRY1N^{150}-R	TTCATCAGTCACTTCCCATGG
	CRY1C^{150}-F	ACTGAACCAACCAGACTCAACC
	CRY1C^{150}-R	CCCGGTTTGTGAAAGCC
	TCP2-TCPn-F	ATGATTGGAGATCTAATGAAGAATAAC
	TCP2-TCP-R	ATGATCTCTTTCCTTGGTCTCTTT
	TCP2-TCPn-F	ATGATTGGAGATCTAATGAAGAATAAC
	TCP2-TCPn-R	TCTTGATAAAGACAAACCAGAAGAG
	TCP2-R&CT-F	TCGGAGCTTAGAGATAAAGCTAGA
	TCP2-R&CT-R	GTTCTTGCCTTTACCCTTATGTT
	TCP2-CT-F	AACCACACTTCGTTTACGGAT
	TCP2-CT-R	GTTCTTGCCTTTACCCTTATGTT
	M13F	GTAAAACGACGGCCAG
	M13R	CAGGAAACAGCTATGAC

	attB-F	GTGGGGACAAGTTTGTACAAAAAAGCAGGCTTC
	attB-R	GTGGGGACCACTTTGTACAAGAAAGCTGGGTC
	TCP2RNAi-1F	GGTGGCATCACAATTCTTCC
	TCP2RNAi-1R	GTCTGATTCTGGTGATTCTCGTC
	TCP2RNAi-2F	AATTCCGGTTCAGATCCGG
	TCP2RNAi-2R	AAACTCTTGATGCTGATGATGAGG
	TCP2RNAi-3F	GTCTCCGCCGCATCAGG
	TCP2RNAi-3R	TCAGTTCTTGCCTTTACCCTTATG
Real time PCR	qACT2-F	GTGGATTCCAGCAGCTTCCAT
	qACT2-R	GCTGAGAGATTCAGATGCCCA
	TCP2-QF	GGGTTTAATTCTCGGGTCG
	TCP2-QR	CGCCGTTGGATTCTGCC
	HYH-QF	GGAACAGAGTATCAGCGCAA
	HYH-QR	CGAAGCATTGTGTTCTCGTT
	HY5-QF	CGG TAG AGA ATC TGG ATC GG
	HY5-QR	CCT CGC TTC CTT TGA CTT TC
	CHS-QF	CAACAGTGAACACATGACCG
	CHS-QR	TGTGGGTTTTCCTTGAGGAA
	CAB-QF	GGGAAACCCTAGCTTGGTCC
	CAB-QR	CCATTTCCTGCGACTCTGTA
	LOX2-QF	AACTACGATTGCATGGGTCA
	LOX2-QR	TCGGTTGGGAAAGTATCCTC
ChIP-qPCR	q-HYH-aF	TTGTGTACCAAAAAATAGAAAACGA
	q-HYH-aR	GCAGACAAAGTCAGAGGCACA
	q-HYH-bF	TTAGTTGATTTTAAGACAAGGGGTC
	q-HYH-bR	CATGAAATGGATCAGAATACTCATG

	q-HYH-cF	GGATTGAAACAGTTGCATTTGTG
	q-HYH-cR	GGTTGAAGAGAATATCAAACCAAAG
	q-HYH-dF	GAGTTGCAGACTTTGAGTGGC
	q-HYH-dR	AGATTTTGCTAAAGACGAGAATAGA
	q-HYH-eF	TCTACAGCTAAACGCCGCC
	q-HYH-eR	TCACCACAGTTTCTTTTTTACCAT
	q-HY5-aF	TCACCTGATCCA AGTCCTGTG
	q-HY5-aR	CATCTCTGTTGCATCACATGA AC
	q-HY5-bF	CTGCCCTAGGCGTGGG
	q-HY5-bR	GATAGGGGAGCTAATTGGGTG
	q-HY5-cF	CTGTCCAAGGACAGGAATGGG
	q-HY5-cR	CAATTGAGATTGGAACGTGGGT
	q-HY5-dF	GGGTTGTTTGGTAATTTTGTGG
	q-HY5-dR	CACTCTGTTTTCCAACTCGCTC
	q-HY5-eF	ATGTGGTGTAGATTCTGAAGAACAC
	q-HY5-eR	CAAGAAGAAGAAGGAGATCAAAGG
	q-HY5-fF	GTGGTTAGCAGCATTGTTCAAG
	q-HY5-fR	GCTTATCTGGAACTCCGTCTTC
ChIP-PCR	HY5-aF	TCACCTGATCCAAGTCCTGTG
	HY5-aR	CTTTTATCTTCGAACCACTTCAAA
	HY5-bF	AGTAGTAGATTCTGTCCAATGGTGT
	HY5-bR	TACGGTGAATAATGGATAGGGG
	HY5-cF	AAAGCACTGCCCTAGGCG
	HY5-cR	AAGAATCGAACGGATATCGAGAG
	HY5-dF	GAATTTACATTTTTACCCTTTATATGG
	HY5-dR	ATGTCTAAGCATCTGGTTCTCGTT
	HY5-eF	AGATTGCAAAGTTGGATCTCTATG
	HY5-eR	GACTACAATAAGAGAACTTGGTCTACA
	HY5-fF	TGTGGTTAGCAGCATTGTTCAA

	HY5-fR	TGGCCCACGCCTAGGG
